# Measuring brain beats: Cardiac‐aligned fast functional magnetic resonance imaging signals

**DOI:** 10.1002/hbm.26128

**Published:** 2022-10-29

**Authors:** Dora Hermes, Hua Wu, Adam B. Kerr, Brian A. Wandell

**Affiliations:** ^1^ Department of Physiology and Biomedical Engineering Mayo Clinic Rochester Minnesota USA; ^2^ Department of Psychology Stanford University Stanford California USA; ^3^ Center for Cognitive and Neurobiological Imaging Stanford University Stanford California USA; ^4^ Department of Electrical Engineering Stanford University Stanford California USA

**Keywords:** cardiac, CSF, fMRI, SMS, vasculature

## Abstract

Blood and cerebrospinal fluid (CSF) pulse and flow throughout the brain, driven by the cardiac cycle. These fluid dynamics, which are essential to healthy brain function, are characterized by several noninvasive magnetic resonance imaging (MRI) methods. Recent developments in fast MRI, specifically simultaneous multislice acquisition methods, provide a new opportunity to rapidly and broadly assess cardiac‐driven flow, including CSF spaces, surface vessels and parenchymal vessels. We use these techniques to assess blood and CSF flow dynamics in brief (3.5 min) scans on a conventional 3 T MRI scanner in five subjects. Cardiac pulses are measured with a photoplethysmography (PPG) on the index finger, along with functional MRI (fMRI) signals in the brain. We, retrospectively, align the fMRI signals to the heartbeat. Highly reliable cardiac‐gated fMRI temporal signals are observed in CSF and blood on the timescale of one heartbeat (test–retest reliability within subjects *R* ^2^ > 50%). In blood vessels, a local minimum is observed following systole. In CSF spaces, the ventricles and subarachnoid spaces have a local maximum following systole instead. Slower resting‐state scans with slice timing, retrospectively, aligned to the cardiac pulse, reveal similar cardiac‐gated responses. The cardiac‐gated measurements estimate the amplitude and phase of fMRI pulsations in the CSF relative to those in the arteries, an estimate of the local intracranial impedance. Cardiac aligned fMRI signals can provide new insights about fluid dynamics or diagnostics for diseases where these dynamics are important.

## Graphical abstract

Blood and cerebrospinal fluid (CSF) pulse and flow throughout the brain, driven by the cardiac cycle. We use rapid functional magnetic resonance imaging (fMRI) measurements to assess blood and CSF flow dynamics in a conventional 3 T magnetic resonance imaging scanner. Cardiac aligned fMRI signals can provide new insights about fluid dynamics or diagnostics for diseases where these dynamics are important.

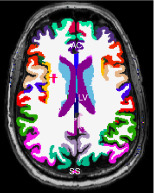

**Abbreviations**
ACA: anterior cerebral artery
CSF: cerebrospinal fluid
FA: flip angle
fMRI: functional magnetic resonance imaging
MCA: middle cerebral artery
MNI: Montreal Neurological Institute
MREG: magnetic resonance encephalography
PC: principal component
PCA: posterior cerebral artery
PPG: photoplethysmography
RMSSD: root mean square of successive differences
SMS: simultaneous multislice
SVD: singular value decomposition
TE: echo time
TR: repetition time

## 1 INTRODUCTION

It is important to understand the connection between the brain's cardiovascular health and cognition. Preclinical studies in aging rodents show that arterial changes may precede cognitive decline (Nation et al., 2019). In COVID‐19, which is primarily thought to affect cardiovascular function, long‐term neuropsychiatric sequelae involving attention and working memory have been observed (Boldrini et al., 2021). The ability to assess cardiovascular efficacy within spatially resolved brain regions, and to connect this assessment to behavior, is also an important direction in magnetic resonance imaging (MRI) research (Wåhlin & Nyberg, 2019). A comprehensive characterization of the status of the brain's fluid dynamics in individual participants can become an important diagnostic tool for cognitive and affective neuroscience. Including this assessment as part of a typical cognitive neuroscience functional MRI (fMRI) experiment may also help to clarify a source of differences between experimental participants.

Blood and cerebrospinal fluid (CSF) pulse through the brain, in synchrony with the cardiac cycle (Womersley, 1955). About each second, a heartbeat produces a pressure wave; blood from the heart traverses the arterial to venous network during multiple heartbeats (Mihara et al., 2003). Various noninvasive MR methods provide specific information about the structure and function of the neurovascular system. MR angiography sequences provide insight into the integrity of the vascular anatomy either by the use of intravenous contrast to enhance blood signal, by designing sequences to manipulate the blood signal relative to be much darker than other tissue (black‐blood), much brighter (bright‐blood) (Nakao et al., 2018), or by introducing flow‐sensitive encoding (phase‐contrast) (Pelc et al., 1991). Arterial spin labeling quantitatively and noninvasively estimates brain perfusion with arterial blood water (Bambach et al., 2020; Telischak et al., 2015).

Other measurements can be cardiac‐gated to assess the dynamic properties of the cardiac pulse. Acquiring one slice at various times after scanning software detects a peak in the heartbeat in real time. In MR elastography, a vibrating source is used during scanning to deform the tissue and estimate its stiffness (Glaser et al., 2012; Kruse et al., 2008; Manduca et al., 2001). A cardiac‐gated MR elastography sequence showed that cerebral vascular compliance, the degree to which tissue absorbs the cardiac pulse, decreases in older individuals (Schrank et al., 2020). Cardiac gated cine MRI methods have also been used to measure dynamic changes in the MR signal (Curtis & Cheng, 2021) and assess the velocity of blood flow in a few slices perpendicular to the major cerebral arteries or the cerebral aqueduct (Enzmann et al., 1994).

These methods are not part of the standard suite of tools used in cognitive neuroscience despite the fact that the spatial distribution of the pulsatile dynamics in blood and CSF spaces change in multiple neurological and neuropsychiatric diseases (Nation et al., 2019; Wåhlin & Nyberg, 2019). For example, aging and Alzheimer's disease alter the rigidity of the arteries, impacting the shape of the flow pulsations. Transcranial Doppler ultrasonography has revealed that increased vascular rigidity increases the pulsatility of the blood flow in the circle of Willis (Roher et al., 2006). Increases in pulsatility with aging may also occur deeper in the microvasculature, which may result in lesions in many brain areas (for reviews, see Tsvetanov et al., 2021; Wåhlin & Nyberg, 2019). In addition, Alzheimer's disease can result in increases in the variability of the fMRI signal (Tuovinen et al., 2020). Variability in resting‐state fMRI signals have been related to cardiovascular dynamics (Bayrak et al., 2021; Chang et al., 2009; Chen et al., 2020; Shmueli et al., 2007) These studies suggest that more detailed information about cardiac pulsations is present in rapidly sampled fMRI data (Tong et al., 2014), but these studies typically focus on understanding the location of the physiological noise in functional data and developing methods to reduce this noise (Frank et al., 2001). Mapping of the pulsatile waveforms of cardiac‐aligned BOLD responses is relevant to understanding disease and for fMRI studies in general, because cardiac pulsations can affect vascular changes in tissue that impact fMRI responses in studies of perception, action and cognition.

The method we describe here builds on these technologies but focuses on a computational approach to extract the waveform shape of the pulsatile cardiac fMRI signal and characterizing its amplitude and timing. Recent development of simultaneous multislice (SMS) methods permits investigators to sample the fMRI signal of the whole brain multiple times within a single cardiac cycle (Larkman et al., 2001; Setsompop et al., 2012). Individual slices can be acquired with a duration of less than 50 ms, and multiple groups of slices are obtained within a single cardiac cycle. Studies measuring this fMRI signal have shown that changes in heart rate can result in changes in the fMRI signal that span several heartbeats (Chang et al., 2009). In single slice acquisitions measured at 7 T, cardiac pulsations could be measured in blood vessels and CSF spaces (Bianciardi et al., 2016; Viessmann et al., 2017). We measure whole‐brain fMRI signals and assess how well the pulse pressure waveform of these signals can be characterized at 3 T. By aggregating measurements over multiple cardiac cycles and, retrospectively, aligning the measurements to the cardiac cycle onset, we obtain a highly reliable noninvasive assessment of the pressure wave and flow of the vasculature and the CSF in many locations across the cranium, that can be well described by a statistical model with two components.

## 2 MATERIALS AND METHODS

In this work, we, retrospectively, align fMRI signals measured at 3 T to the cardiac pulse using a widely used SMS protocol. In this sequence, forty slices are measured in five groups, each group comprising eight separated planes. The data in a single group of planes are acquired simultaneously over a 50 ms interval; the entire brain—all five groups—is measured every 250 ms. The typical cardiac cycle duration is about 1000 ms. The measurements cover the whole‐brain and provide spatially‐resolved information of 4 mm isotropic voxels. We compared these responses to fMRI responses measured during a typical resting‐state fMRI scan (TR = 2 s, 240 volumes). Even within a single‐subject, the cardiac‐aligned time series modulations can be very reliable when splitting data in half across the 3.5 min scan. Within subjects, the most reliable cardiac aligned modulations are located near the principal arteries and veins, portions of the CSF, and certain gray matter regions. Blood vessels showed local minima around the time of the PPG peak while CSF spaces showed local maxima. These response features were consistent across the SMS and slow fMRI sequence. This article describes the measurements, analytical methods and signals in healthy subjects.

### 2.1 Subjects and IRB statement

Data from 10 healthy subjects are analyzed in the study. The study was approved by the Stanford University Institutional Review Board and all subjects provided informed consent to participate in the study. Five subjects (age 24–63 years old, two males) were recruited specifically for measurements using fast SMS methods. One of these subjects was scanned twice, separated by a 3‐year interval. We analyzed additional data from five subjects (age 30–60 years old, three males) who were part of a different study that obtained resting‐state data as a control condition (Hack et al., 2021). The acquisition parameters for both sets of subjects are described below.

### 2.2 Anatomical MRI

All subjects were scanned on a 3 T General Electric MRI 750 scanner at the Stanford Center for Cognitive and Neurobiological Imaging. In order to localize different anatomical gray matter regions and CSF spaces, we acquired a T1‐weighted anatomical image (1 × 1 × 1 mm voxels). We segmented the T1 weighted scan using Freesurfer (http://surfer.nmr.mgh.harvard.edu/, (Fischl, 2012)). In order to understand the effects of the arterial pulsations, we assigned each gray matter region from the Desikan‐Killiany Atlas (Desikan et al., 2006) to a “distance” level varying in four steps from closest to furthest from one of the main three arterial branches: the anterior cerebral artery (ACA), middle cerebral artery (MCA), and posterior cerebral artery (PCA). To map veins, we collected an MR venogram (0.43 × 0.43 × 2 mm voxels) and thresholding this image allowed us to select the superior sagittal sinus.

In order to compare the location of reliable cardiac averaged responses across subjects, the nonlinear transformation to MNI152 space was calculated based on the T1 scan using unified segmentation in SPM12 (Ashburner & Friston, 2005).

### 2.3 Cardiac cycle measurements using PPG

**FIGURE 1:**
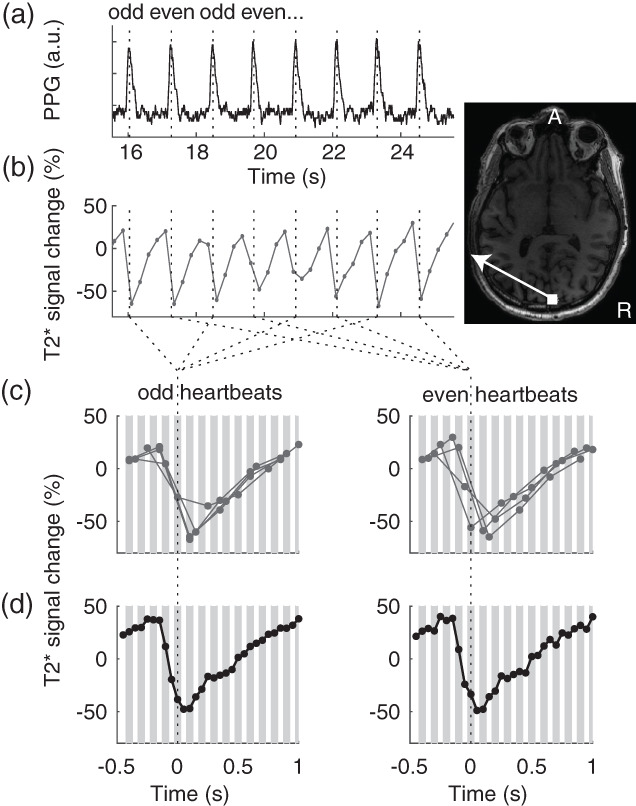
Temporal alignment of magnetic resonance imaging (MRI) measurements to heartbeat. (a) The photoplethysmography (PPG) signal was measured with a pulse oximeter. Peaks in the signal were detected (dotted lines). (b) The functional magnetic resonance imaging (fMRI) signal from a voxel near the superior sagittal sinus was measured every 250 ms, for a duration of 50 ms. (c) Signals were aligned to even and odd heartbeat peaks, gray lines indicate the 50 ms within which each time‐point was measured. This example shows the signals from the eight heartbeats in (b). (d) The signals averaged for all even and odd heartbeats form a smooth curve of the cardiac‐gated response, sampled every 50 ms.

To estimate the cardiac cycle a pulse oximeter was attached to a finger and the photoplethysmography (PPG) signal was measured during MRI scanning (Figure 1a). Peak detection was performed using custom code in MATLAB (The MathWorks, Inc.), which is shared on our Github webpage (the physioGet.m function in https://github.com/vistalab/BrainBeat). In this code, the peak in the autocorrelation of the PPG signal is detected to reveal the heart rate, such that peak detection (using MATLAB's findpeaks function) could be done with a minimum peak distance of 70% of the heartbeat cycle. Before detecting the peaks, PPG data were low‐pass filtered at 5 Hz using a third‐order Butterworth filter in two directions. This allowed the detection of the peaks in the PPG signal (known to be related to systole) that occurred around every heartbeat cycle.

We found this method to be overall more reliable compared to the peaks detected by the scanner software (Supplemental Figure 1). We also compared the detection of peaks in the PPG signal to the peaks detected in the ECG signal that was simultaneously measured in two of the subjects (Supplemental Figure 2), and find that, while the ECG signals suffer from relatively more contamination of scanner noise, there was little variability across the relative differences between the PPG and ECG peaks, indicating that the timing of the PPG peaks show little beat to beat variability.

To ensure that there were no large within subject variations in heart rate during the scans, we calculated heart rate variability. Since the scans only had a duration of 220 s, we quantified heart rate variability across ultrashort intervals (20 s) as in Salahuddin et al. (2007), using the root mean square of successive differences (Supplemental Figure 3). If the heart rate variability was typically less than a slice acquisition duration (50 ms), no further within scan corrections were deemed necessary. Supplemental Figure 6 further shows that the respiratory rate was quite stable within subjects across the scan.

### 2.4 Functional MRI

In five subjects, we acquired SMS, fMRI measurements to map brain wide cardiac‐gated fMRI signal variations. Whole‐brain fMRI data were acquired using several gradient echo EPI sequences with whole brain coverage and SMS (sometimes also called multiband or hyperband) (Larkman et al., 2001; Setsompop et al., 2012). First, an SMS sequence was used with 4 mm isotropic voxels, a flip angle of 48°, TR = 250 ms, TE = 11.6 ms, FOV = 224 × 224 and 40 slices with multiband factor 8. This resulted in a slice acquisition time of 50 ms. A total of 878 volumes were acquired within a total scan duration of 220 s.

In five additional subjects, we analyzed data from a typical resting‐state fMRI study (Hack et al., 2021) where PPG signals were acquired. A whole brain EPI sequence was used with 3 mm isotropic voxels, a flip angle of 77°, TR = 2 s, TE = 27.5 ms, FOV = 216 × 216 and 45 slices. A total of 240 volumes were acquired within a total scan duration of 480 s.

Every slice is acquired at a different time with respect to the cardiac pulse, and we wanted to preserve the 50 ms (SMS) and 44.4 ms (resting state) sampling per slice. It was therefore essential to preserve the measured voxel time series and not perform slice timing correction. The fMRI data were inspected for motion and no data were excluded for motion. The linear transformation matrix to spatially align the fMRI with the anatomical data were calculated to align the functional volumes.

### 2.5 Calculation of cardiac aligned responses

We calculated cardiac averaged responses as follows. The first three volumes were removed. The remaining time measurements were transformed to units of percent modulation and linear trends were removed (Figure 1b). For every voxel in each slice, the slice time was noted with respect to the heartbeat peaks on the PPG signal measured on a finger (Figure 1c). Each slice was measured within a 50 ms temporal window, and the average PPG‐peak‐aligned signal was calculated pooling from the entire acquisition (Figure 1d). Given that heartbeats are spaced about 1 s duration, there are about 20 sample windows between heartbeats.

To calculate reliability of these cardiac‐gated responses, we averaged signals separately from the even and odd PPG peaks (Figure 1d). We then calculated the coefficient of determination between even and odd heartbeats. The coefficient of determination (*R* ^2^) is a standard statistical measure of the reliability with a maximum of 100, that is here used to quantify the reliability of the cardiac‐gated time series.

$$R^{2}=100*\left(1-\frac{{\mathrm{SS}}_{\text{residuals}}}{{\mathrm{SS}}_{\text{data}}}\right)$$

$${\mathrm{SS}}_{\text{residuals}}=\sum_{i}{\left(y_{i}-f_{i}\right)}^{2}$$

$${\mathrm{SS}}_{\text{data}}=\sum_{i}{\left(y_{i}\right)}^{2}$$

where *y*_*i*_ is the even response amplitude and *f*_*i*_ is the odd response amplitude for time point *i*. Note that *R* ^2^ is defined here with respect to zero, rather than with respect to the mean response, to avoid the arbitrariness of the mean.

For visualization purposes, results in individual subjects are displayed with *R* ^2^ > 50. When averages across the subjects are calculated for voxels converted without smoothing to MNI152 space, results are displayed for average *R* ^2^ > 30.

### 2.6 Statistical modeling of cardiac averaged responses

Capturing the amplitude and temporal delay of the cardiac pulsatility in different cranial compartments may be important to further understand disease mechanisms. In order to quantify the temporal delay and amplitude of the responses, we developed a parameterized model of these waveforms using singular value decomposition (SVD) based on the SMS time series time series data. The amplitude of the temporal waveforms of the cardiac averaged responses in each voxel was set to the *R* ^2^, such that the largest responses entering the SVD are the ones that are the most reliable across the cardiac cycle, and the smallest responses were those that are the least reliable. This results in a matrix *M* of dimensions voxels by time, which was decomposed using SVD: $M=\mathrm{U\sum}V^{*}$. The columns of *U* contain the eigenvectors as a function of time, and *V* contains the spatial weighting for these vectors across the brain. The SVD was performed on half the data from the odd heartbeats. We then calculated the number of components that explained over 70% of variance in the other half of the data from the even heartbeats.

We used the following strategy to develop a model across the subjects. The principal components were calculated in each individual subject as a function of time. To compare waveforms across subjects, waveforms were normalized in time and resampled across the heartbeat cycle to create standardized responses as a function of heartbeat cycle. A second SVD was then done on these components, leaving one subject out for cross‐validation, resulting in a set of canonical principal components. A weighted combination of these canonical components could then be used to predict the cardiac aligned responses from the odd heartbeats in the left out subject $\left(Y=\beta_{1}{\mathrm{pc}}_{1}+\ldots +\beta_{n}pc_{n}+\epsilon \right)$. We then calculated the relative root mean squared error ($R_{\text{rmse}}$), for each voxel. The $R_{\text{rmse}}$ describes how well the model (fitted on the training subjects) explained the cardiac averaged even heartbeat responses in the test subject compared to the test–retest reliability and is defined as $R_{\text{rmse}}=\frac{M_{\text{rmse}}}{D_{\text{rmse}}}$, where $M_{\text{rmse}}$ is the model prediction error and $D_{\text{rmse}}$ is the data prediction error between even and odd responses. If $R_{\text{rmse}}<1$, the model fitted on the other subjects predicts the data better compared to the within subject test–retest reliability. If a model perfectly predicts a signal with zero‐mean Gaussian noise and standard deviation σ, the expected value of the $R_{\text{rmse}}$ is $\frac{1}{\sqrt{2}}$. This model‐based approach reduces noise in the waveform. For voxels with reliable waveforms (with *R* ^2^ in individual subjects larger than 50%), a well‐fitted model thus allows a reliable characterization of the times of the local minima and maxima of the cardiac aligned responses.

## 3 RESULTS

In order to map brain‐wide cardiac pulsations, we used a rapid fMRI acquisition and, retrospectively, calculated cardiac aligned responses.

### 3.1 Regions with reliable cardiac aligned responses

**FIGURE 2:**
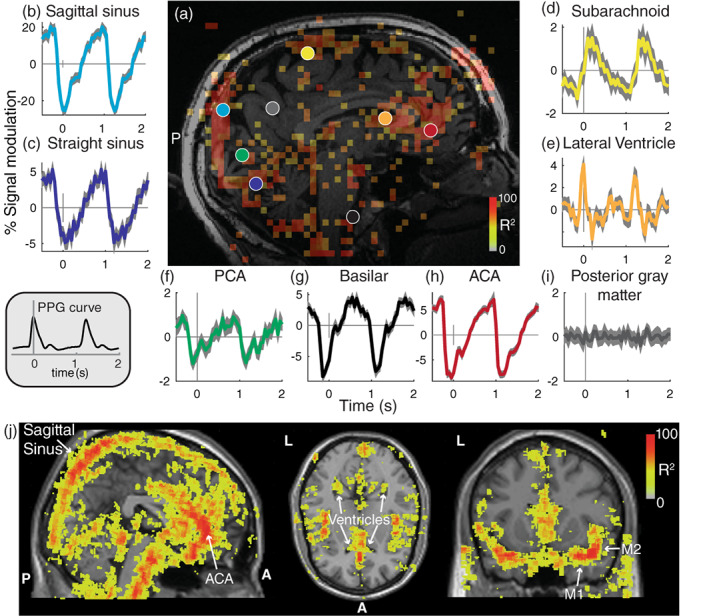
Reliable cardiac‐aligned responses in a single subject. (a) Sagittal slice showing voxels that had highly reliable cardiac aligned responses (*R* ^2^ > 50). The colored circles indicate locations of the responses in panels (b–i). The average (±2*SE) cardiac aligned time series are plotted; zero indicates the peak in the photoplethysmography (PPG) responses (gray inset). Cardiac aligned responses are shown for the sagittal sinus (b), straight sinus (c), subarachnoid (d), lateral ventricle (e), posterior cerebral artery (f), basilar artery (g), anterior cerebral artery (h), and posterior gray matter (i). (j) Across all subjects, a map of the reliability of the cardiac aligned responses represented in MNI152 space (without smoothing). Colors overlaid on a standard MNI brain indicate the average reliability (*R* ^2^) across subjects for a sagittal, axial and coronal slice. Maps are thresholded and only voxels with an average *R* ^2^ > 30 are shown to highlight those areas with reliable modulations. The sagittal sinus, ventricles, anterior cerebral artery (ACA), and M1 and M2 segments of the middle cerebral artery (MCA) are labeled

Several brain regions have reliable responses at the frequency of the cardiac cycle in an individual subject (Figure 2a) and across the group (Figure 2j) not all voxels have reliable cardiac aligned responses. First, voxels close to the sagittal and straight sinus show reliable responses. In the individual subject, these responses show a local minimum around the same time as the peak of the PPG curve (time zero for cardiac aligned responses) (Figure 2b,c). Second, voxels close to the PCA, basilar artery, and ACA similarly show reliable responses with a local minimum 0.1–0.2 s earlier compared to the PPG peak (Figure 2f–h). Third, voxels near CSF regions such as subarachnoid spaces and the lateral ventricle show reliable responses with a local maximum around the same time as the PPG peak (Figure 2d,e). Importantly, not all brain regions show large cardiac pulsations, such as an area in the posterior gray matter that is distant from arteries, veins, and CSF spaces (Figure 2i).

### 3.2 Regional characterization of the cardiac aligned response shape

**FIGURE 3:**
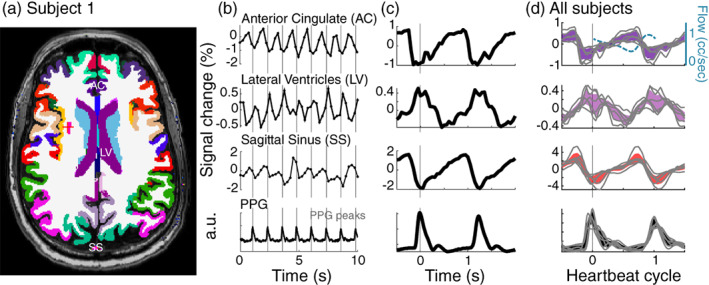
Reliable response shapes across subjects in several anatomical regions. (a) Example slice with gray matter segmentation from Freesurfer, showing the anterior cingulate (AC), the lateral ventricle (LV), and the sagittal sinus (SS) segmented from the venogram. (b) Functional magnetic resonance imaging (MRI) waveforms averaged across these three segmented areas, sampled every 250 ms. (c) Aligning samples to each photoplethysmography (PPG) peak allows a clear visualization of the waveform of the cardiac aligned responses within these regions of interest. (d) Average curves for all six subjects when resampled as a function of the heartbeat cycle in each subject (mean ± 2*SE). Gray lines show consistent shapes of cardiac aligned curves in each subject. The top panel also includes a curve of the typical flow speed (cc/s) in the carotid artery (dashed light blue), this curve is based on the literature where speed was measured using cine phase contrast MRI (Enzmann et al., 1994; Wagshul et al., 2011)

To understand whether the waveform of the cardiac aligned responses seen in Figure 2 is reliable across subjects, we segmented the T1 anatomical scan into several anatomical regions (Figure 3a). Figure 3b shows that reliable cardiac signals are observed in the raw fMRI signal from voxels located at the anterior cingulate (a brain region close to the ACA), the lateral ventricles and the superior sagittal sinus. The anterior cingulate waveform has a local minimum before the time of the PPG peak. This is in line with the fact that the PPG peak is measured on the thumb, and blood typically arrives in the brain before it arrives in the hand. The sagittal sinus waveform has a local minimum at a similar time as the PPG peak. We note that these waveforms are opposite from blood flow measurements in the carotid artery reported in the literature (Enzmann et al., 1994; Wagshul et al., 2011) show a sharp rise in the speed of flow related to systole that aligns with the sharp drop in our data (Figure 3d, top panel, dashed blue trace). In contrast with the blood vessels, the lateral ventricles show local maxima at the time of the PPG peak. These response shapes are highly robust across the subjects (Figure 3d).

### 3.3 Cerebral arteries influence the pulsatility in the gray matter

**FIGURE 4:**
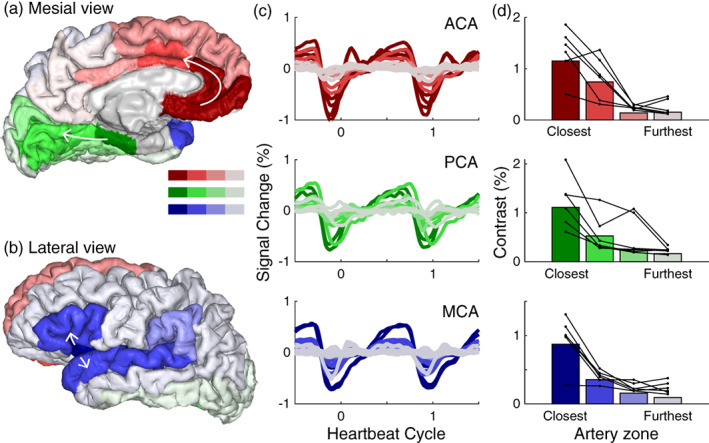
Response modulation in gray matter decreases with distance from the input to the main arteries. (a) Mesial and (b) lateral view of a cortex rendering segmented using Freesurfer and labeled according to the position on the three main arterial zones: The anterior cerebral artery (ACA) (red), posterior cerebral artery (PCA) (green), and middle cerebral artery (MCA) (blue). (c) Average cardiac aligned responses across subjects during the heartbeat cycle in these three arterial zones. Lighter colors (gray) indicate areas further along each branch and typically show a decrease in modulation. (d) The modulation amplitude within these areas averaged across subjects (bars), and for each subject (connected black lines/dots). There is one subject in each row of (d) with the lowest contrast in the closest arterial zone; this is the same subject across all plots

While Figure 3 illustrates reliable cardiac pulsations in the gray matter of the anterior cingulate, Figure 2 shows that many other gray matter regions do not have reliable cardiac pulsations. We therefore consider that the brain's blood supply comes from three branches of the cerebral arteries; the posterior, anterior, and middle cerebral arteries (PCA, ACA, and MCA). We hypothesize that the cardiac aligned responses in the gray matter will be large near the inputs to these three arteries. To measure this, we combined gray matter areas, segmented with Freesurfer, into three groups (Figure 4a,b) that are supplied by the three main cerebral arteries. Within each group, we assign an area to a subgroup depending on its distance from the main inputs. The rostral anterior cingulate and medial orbitofrontal cortex were closest to the main input from the ACA, the parahippocampal gyrus was closest to the PCA and the insular cortex and transverse temporal gyrus were closest to the MCA. Figure 4c shows that there is a decrease in pulsatility along the three main arteries: areas closest to the main arterial inputs have the largest cardiac averaged pulsations and more distant areas have smaller cardiac pulsations. This decrease in the amplitude of the cardiac aligned response with distance from the main input of each cerebral artery is reliably observed across subjects (Figure 4d). Moreover, the gray matter areas close to the main inputs of the three arteries also show similar response waveforms, with local minima before the PPG peak (time zero).

### 3.4 Timing of blood and CSF pulsations

The analysis of the fMRI time series shows that cranial spaces with blood and CSF have reliable cardiac aligned responses. Areas close to the inputs to the cerebral arteries show local minima close to the time of the PPG peak, and the ventricles show local maxima. The timing of the cardiac aligned responses provides information about the speed of the pulse pressure wave and can quantify delays between the arteries and veins. Pulse delays between arteries and veins could indicate the time that it takes for the cardiac pulse pressure waves to travel through the vascular system. Pulse wave velocity is related to arterial stiffening and pulse wave velocity measured between carotid and femoral arteries has been associated with an increased risk to develop dementia (Rouch et al., 2018). We therefore test whether the cardiac aligned responses allow us to map the timing of the pulse pressure wave.

**FIGURE 5:**
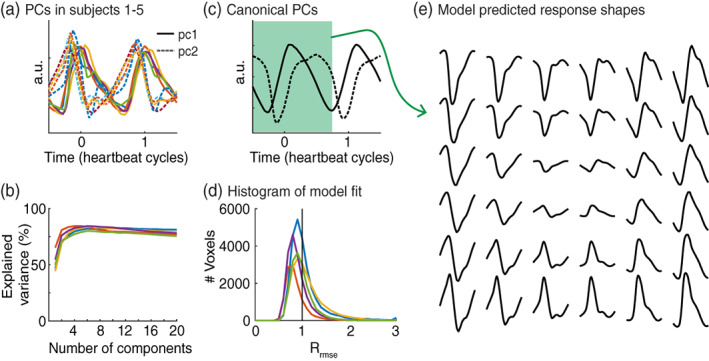
Model of the cardiac aligned responses. (a) First, two principal components (PCs) for all five subjects plotted as a function of the heartbeat cycle. Time zero is the photoplethysmography (PPG) peak. The first principal component is plotted in the solid line; the second component is plotted as a dashed line. (b) The cross‐validated variance explained by an increasing number of principal components plotted for each subject. (c) Canonical principal components across subjects as a function of heartbeat cycle. (d) Histogram showing the distribution of the relative root mean square errors across voxels. A *R*_mse_ < 1 indicates that the model trained on four out of five subjects explained half of the data in the fifth subject better compared to the other half of the data from the same subject. Note that the expected value of a perfect model and Gaussian measurement noise lies around $\frac{1}{\sqrt{2}}$. (e) The model with these two principal components can predict various response shapes, the waveforms show time ranging from −0.50 to 0.66 of the heartbeat cycle (indicated in green in (c))

To extract an image with the timing of the cardiac aligned responses, we perform a SVD to clean the waveforms (Figure 5a). We do this based on half the data, such that the validity of the model can be tested using the other half of the data. Two principal components explain >68% of variance in the data (Figure 5b). The shape of these two components is highly consistent across subjects and allows extracting two group level canonical principal components (Figure 5c). The model with two canonical principal components fitted with cross validation (leave‐one‐subject‐out) explains the data from the left out subject well (Figure 5d). Moreover, this model‐based approach does not rely on a reliable signal from a particular region of interest or artery to calculate pulse timing, but provides a set of canonical curves that can be regressed against all data to extract the waveform shape and timing. The relative root mean squared error ($R_{\text{rmse}}$) was smaller than 1 in 46.5–83% of voxels (range across subjects), indicating that the model with two components explains the data from the left out subject better than within subject test–retest reliability. The leave‐one‐out cross validation suggests that the model will perform well in other subjects. A model based on these two canonical principal components describe various possible responses (Figure 5e and Supplemental Figure 4).

**FIGURE 6:**
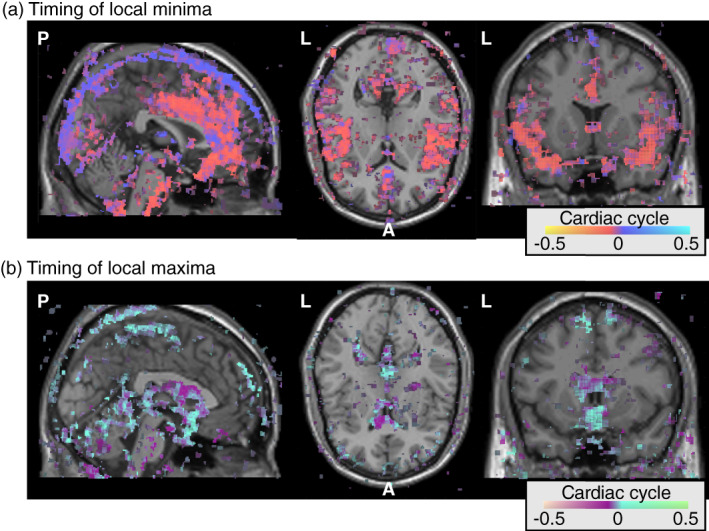
Spatial distribution of the timing of cardiac aligned responses in the cranium across the group. For each voxel in each subject, we calculated the peak time of the cardiac aligned response in terms of the cardiac cycle with the peak of the photoplethysmography (PPG) at time zero. These timing maps were aligned in MNI152 space; in this way, the average timing across the subjects could be calculated. (a) The timing of the *local minima* for all voxels that had an average *R* ^2^ > 30 across the subjects. Red colors indicate times before the PPG peak and blue colors indicate times after the PPG peak. (b) The timing of the *local maxima* for all voxels that had an average *R* ^2^ > 30 across the subjects. Purple colors indicate times before the PPG peak and cyan colors indicate times after the PPG peak

This model‐based approach reduces noise and reliably characterizes the times of the local minima and maxima of the cardiac aligned responses. Figure 6a shows the average times of the local minima across subjects in terms of the cardiac cycle. Areas close to the main cerebral arteries such as the anterior cingulate cortex and the insula show pulses with a local minimum before the PPG peak (red/yellow). The superior sagittal sinus shows a later time on the local minimum right after the PPG peak (blue). Interestingly, these peaks are relatively close in time, separated only by about 20% of the cardiac cycle, which corresponds to ~200 ms at a heart rate of 60 beats per minute.

Figure 6b shows the average times of the local maxima across subjects. Across subjects, the ventricles show the earliest times with a local maximum before the PPG peak (purple). Other areas with CSF, such as the subarachnoid cisterns and subarachnoid spaces under the superior sagittal sinus, show later pulses with a local maximum after the PPG peak (cyan). Similar as in the blood vessels, the difference in timing of the local maxima across the different CSF spaces is small, separated only by about 20% of the cardiac cycle.

**FIGURE 7:**
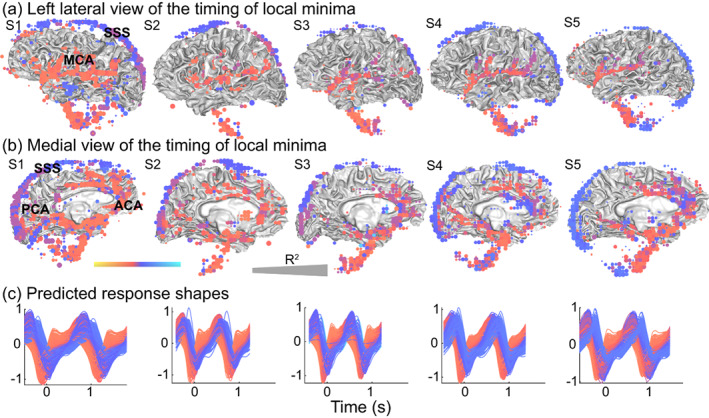
Distribution and timing of the local minima in the cranium of individual subjects. For all subjects (S1–S5), we calculated the timing of the local minima of the modeled response in terms of the cardiac cycle for each voxel, with the peak in the photoplethysmography (PPG) located at zero. (a) Left lateral view of times of local minima in voxels with reliable cardiac aligned responses (*R* ^2^ > 50) were rendered on the gray/white matter surface. Red colors indicate an earlier minimum such as seen in areas close to the middle cerebral artery (MCA), and blue colors indicate a later minimum such as seen in the superior sagittal sinus (SSS). Larger dots indicate that voxels were more reliable, with the largest dots reflecting *R* ^2^ = 100. (b) Medial view of the times of the same local minima, red colors show earlier minima in areas close to the anterior cerebral artery (ACA), and posterior cerebral artery (PCA) and blue colors show later peaks such as those in the superior sagittal sinus (SSS). (c) Predicted cardiac aligned response shapes for all displayed voxels as a function of time (s). Colors match the color of the voxels. The following supplemental movies show the dynamic evolution of these cardiac responses Movies S1 and S2

This general pattern of response timing is also observed in individual subjects. Similar to the group average, individual subjects show early local minima in regions near the main three cerebral arteries and later local minima around the superior sagittal sinus (Figure 7a,b). Note that there are also significant local minima underneath the rendered cortex that likely correspond to the basilar and carotid arteries. Figure 7c shows the full waveforms as a function of time in seconds, where colors correspond to rendered voxels. By showing the waveforms as a function of time, the relative peaks of the compartments can be more easily interpreted. While subject 2 had a much faster heart rate compared to subject 1, both subjects show responses in the arteries that peak about 200 ms earlier (red) compared to responses in the veins (blue). The evolution of the pulsations can also be seen in the Supplemental Movies (Movies S1 and S2).

**FIGURE 8:**
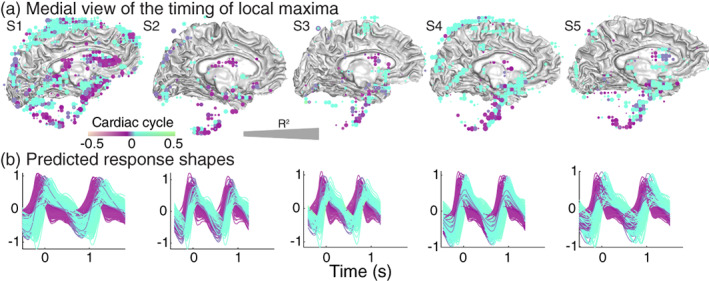
Distribution and timing of the local maxima in the cranium of individual subjects. For all subjects (S1–S5), we calculated the timing of the local maximum of the modeled response in terms of the cardiac cycle for each voxel, with the peak in the photoplethysmography (PPG) located at time zero. (a) Medial view of times of local maxima in voxels with reliable cardiac aligned responses (*R* ^2^ > 50) were rendered on the gray/white matter surface. Purple colors indicate peak times before the PPG peak and cyan colors indicate times after the PPG peak. (b) Predicted cardiac aligned response shapes for all displayed voxels as a function of time (s). Colors match the color of the voxels. The following supplemental movies show the dynamic evolution of these cardiac responses Movies S1 and S2

Similar to the average across the subjects, individual subjects also show early local maxima in areas near the ventricles and later local maxima in areas near the subarachnoid cisterns (Figure 8). Note that there are also significant local maxima underneath the rendered cortex that likely correspond to the CSF spaces near the fourth ventricle. However, there are interindividual differences in the locations that show a local maximum in the reliable cardiac pulsation at later times. Note that the age range for the subjects was 24–63 years old. Figure 8b shows the full waveforms as a function of time in seconds, where colors correspond to rendered voxels. By showing the waveforms as a function of time, the relative peaks of the compartments can be more easily interpreted. The evolution of the pulsations can also be seen in the Supplemental Movies (Movies S1 and S2).

### 3.5 Cardiac‐gated resting state

**FIGURE 9:**
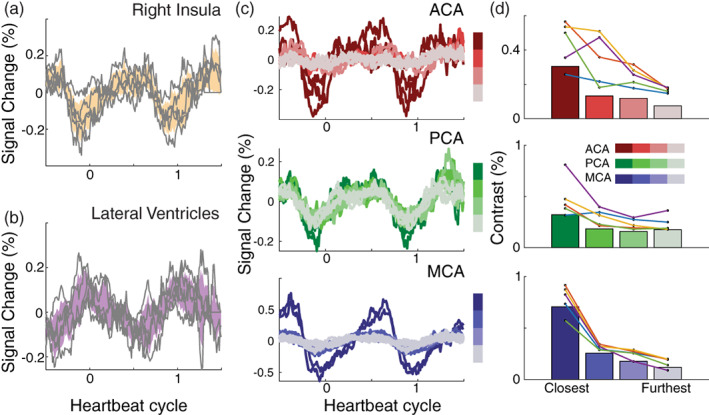
Cardiac aligned responses in resting‐state functional magnetic resonance imaging (fMRI) data. From a typical resting‐state fMRI scan, we extracted cardiac aligned responses. (a) The mean cardiac aligned response in the insula for each subject in gray lines and average ±2 standard errors in yellow. (b) The mean cardiac aligned response in the lateral ventricles for each subject in gray lines and average ±2 standard errors in purple. (c) Average brain pulsations across subjects as a function of heartbeat cycle along these three arterial zoned anterior cerebral artery (ACA) (red, top); posterior cerebral artery (PCA) (green, middle); and middle cerebral artery (MCA) (blue, bottom). Lighter colors (gray) indicate areas further along each branch and typically show a decrease in pulsatility. (d) Contrast within these areas averaged across subjects (bars), and for each subject (connected colored lines/dots)

In order to understand whether the characteristic blood and CSF pulsations could also be observed with a typical resting‐state fMRI scan, we analyze data from five subjects who were scanned in a different project (Hack et al., 2021). In these resting‐state scans, the echo time is slower and the repetition time is slower (SMS: TE = 11.6 ms, FA = 48, TR = 250 ms, resting‐state fMRI: TE = 27.5 ms, FA = 77, TR = 2000 ms), but each slice is still acquired within a short time of 44.4 ms. Each slice can be aligned to the PPG peak, and cardiac pulsations can be extracted. Similar to the fast SMS sequence, areas near main cerebral arteries, such as the left insula (Figure 9a) show large cardiac pulsations with a local minimum. Voxels in the lateral ventricles also show large cardiac pulsations, with a local maximum as in the SMS data (Figure 9b compared to Figure 3c,d). Similarly, the amplitude of the cardiac pulsations in the gray matter decreases with distance from the main three cerebral arteries (Figure 9c,d).

The fact that cardiac alignment of the resting‐state fMRI results in similar response curves indicates that these responses are robust despite differences in scan sequences. Using the gradient echo signal equations (Hornak, 1996), we calculate that the SMS sequence has a higher sensitivity to changes in T1 than the resting‐state sequence, but slightly lower sensitivity to changes in T2*. Given the larger modulation measured using SMS, we believe the signal modulation is T1‐weighted, likely carried by increases and decreases of the blood volume.

We note that the time in which one slice is acquired is comparable between the two methods: 50 ms for the SMS sequence and 44.4 ms for the resting‐state sequence. Much longer slice acquisition times would likely blur the cardiac pulsations. While cardiac aligned responses overall have similar characteristics, it has to be noted that there is more noise in the regular resting‐state acquisition compared to fast SMS acquisition. The resting‐state fMRI scan is about 2× longer and each slice is only acquired once every 2 s, while a slice is measured four times per second for the SMS data. Acquisition of fewer measurement time points per slice using an SMS sequence would also result in less reliable characterization of the cardiac pulsations (Supplemental Figure 5).

## 4 DISCUSSION

The SMS technique allows a rapid assessment of cardiac pulsations in arteries, veins, and CSF. These pulsations produce a local minimum after systole in the fMRI signal in arteries and veins, and a local maximum in CSF spaces (Figures 2, 3, 4, and 6, 7, 8). The timing of these extrema matches known physiological dynamics in blood and CSF (Figure 3d). Early local minima are observed in gray matter regions near the three cerebral arteries (Figure 4) and later local minima are observed in the veins (Figures 6 and 7). Early local maxima are observed in the ventricles and later local maxima are observed in the subarachnoid spaces (Figures 6 and 8), with the latter having more spatial variability across subjects. An analysis of resting‐state fMRI data acquired with a conventional sequential slice acquisition shows that these physiological signatures are not unique to the SMS sequence (Figure 9), but are a general property of fMRI signals in blood and CSF.

### 4.1 Physiological basis of the cardiac‐gated MR signal

The fMRI signal measures spin‐coherence; a number of physiological properties jointly determine the signal level. By retrospective cardiac alignment, we obtain a temporal response that informs us about physiological signals that are principally driven by the beating heart. The signal analysis suggests that we are measuring two independent parameters: a signal amplitude and phase. Consequently, we have limited ability to make inferences about the many different factors that can give rise to the cardiac‐gated fMRI signal (proton density, blood velocity, vessel stiffness) and how the signal depends on imaging parameters (TE, TR, FA, voxel size).

There has been some initial theory exploring the relationship between these factors for high field (7 T) measurements that acquire a single fMRI slice (Bianciardi et al., 2016; Viessmann et al., 2017, 2019). An important difference between these single slice 7 T measurements and the whole brain measurements in our current study is that inflow and velocity influence the signals in a very different manner. When measuring a single slice, inflowing spins have not experienced prior excitation and signals follow a set of relatively well worked out equations (Bianciardi et al., 2016). The same equations may not apply to a whole brain acquisition. An example of the size of these effects can be found in a study measuring fMRI fluctuations on the timescale of seconds during sleep. The CSF in the fourth ventricle measured in the bottom slice of the acquisition showed large signal increases due to inflow at the same time as the fMRI signal in gray matter decreased (Fultz et al., 2019). These CSF signal increases were suppressed in slices that were not at the edge of the acquisition. While we characterize the temporal response shapes, it will be important to develop a better understanding of how whole brain fMRI signals are affected by different physiological parameters that fluctuate during the cardiac cycle such as blood or CSF volume, flow speed and partial voluming.

The effect of physiological parameters, such as blood flow speed, volume, and pressure vary across the different compartments (arteries, veins, sinuses, CSF). As an example of the complex interaction of these factors, notice that arteries, but not veins, pulsate (expand) during the heartbeat. Even so the venous compartments, such as the superior sagittal sinus, have a large transient wave in the fMRI signal. This is because the fMRI signal does not depend uniquely on any of these physiological factors, but rather captures a change in the local magnetic field, largely T1‐weighted. As a further complexity, the average speed of flow in the superior sagittal sinus is about 13 cm/s (range ~4–20 cm/s) (Jordan et al., 1994), much lower than the speed of flow of 40 cm/s (range ~20–55 cm/s) in the carotid (Enzmann et al., 1994; Enzmann & Pelc, 1991). However, variations in blood flow volume across the cardiac cycle vary from 140 to 463 ml/min in the superior sagittal sinus (Jordan et al., 1994), which is much higher compared to cardiac cycle variations of 200–350 ml/min in a unilateral carotid artery (Enzmann et al., 1994). The causes of the measured waveforms thus likely differ between the arteries and the veins. The measured waveform in the arteries likely have a strong contribution by changes in the speed of flow, while the measured waveforms in the veins likely have a stronger contribution from changes in blood flow volume. Flow velocities are much lower again in the CSF and CSF velocities in the cervical subarachnoid space vary from −2 to 2 cm/s across the cardiac cycle (Enzmann & Pelc, 1991). These parameters all influence the fMRI signal. Thus, while some sequences aim to isolate a single physiological factor, such as flow speed, the fMRI signal provides a relatively comprehensive view.

We find local minima in blood vessels around the time of the PPG peak. The local minimum is defined by a rapid decline when the cardiac pulse arrives, followed by a slow increase. The opposite pattern, with a local maximum, was observed in CSF spaces. These time courses were consistent between the fast SMS measurements and the resting‐state fMRI measurements. Another group, who defined a new whole brain magnetic resonance encephalography (MREG) sequence showed similar dips in the MREG signal (filtered for cardiac frequencies) in the ACA following systole (Rajna et al., 2021), consistent with prior reports using multi‐slab echo‐volumar imaging (Posse et al., 2013). Rather than relying on local minima and maxima, we use an approach based on the complete waveform. We create a model with two principal components, and the relative weights on these components differentiate the local minima in the vascular spaces from local maxima in the CSF (Figures 6, 7, 8, Supplemental Figure 4b). This segregation is in line with cranial fluid dynamics that show that the cardiac pulse has opposite effects on blood and CSF (Wagshul et al., 2006). During the heartbeat, blood moves in and occupies a larger volume, and CSF moves out and occupies a smaller volume (Beggs, 2014). This explains why the time course in blood and CSF would be inverted.

### 4.2 Complementary nature of various MRI‐based measurements of cardiac pulsations

The pulsatile flow during the cardiac cycle is accompanied by several mechanical and physiological factors that can be assessed in various complementary ways. Cine MRI measurements encoded for displacements have shown that some brain areas move about 0.1 mm during the cardiac cycle (Soellinger et al., 2009). Amplified MRI analyses represent these cardiac‐gated signals as movements of the ventricles, blood vessels and at gray or white matter boundaries (Abderezaei et al., 2020; Kolipaka et al., 2021; Terem et al., 2018).

Further understanding the relation between the pulsatile motion and fluid dynamics is important. Pial blood vessels are surrounded by CSF (Iliff et al., 2013). One study measured cardiac cycle induced arterial wall motions (Mestre et al., 2018) using particle tracking velocimetry and two photon imaging through a sealed cranial window in mice. Arterial diameters were about 10 μm and increased with about 0.1 μm during the cardiac cycle. Average arterial diameters did not change during hypertension, but the typical vessel wall speeds were increased in hypertension, in particular in more distal arteries. These hypertension related increases in wall motion were further accompanied by a reduced forward CSF flow in surrounding CSF spaces. The use of complementary techniques may further elucidate different physiological factors noninvasively in humans in neurological and neuropsychiatric diseases.

### 4.3 Retrospective cardiac alignment reveals temporal waveforms of the cardiac response

While our data have a relatively low spatial resolution with 4 mm isotropic voxels, our measurements have the advantage that with a brief fMRI scan, they reveal detailed temporal profiles of the pulsations in blood and CSF spaces. We, retrospectively, aligned the measured signals to the PPG peak, which allowed us to observe that the cardiac pulsations are not sinusoidal, with a steep slope when the pulse arrives followed by a shallow slope on return. A spectral approach would not have been sensitive to these characteristics of the pulsatile waveform, which have been related to fluid dynamics. For example, the slope and amplitude of the pulse pressure wave may be related to intracranial compliance, with a less compliant tissue resulting in a steeper slope (Wagshul et al., 2011). A study in an older population used a high resolution 4D flow MRI measurement with velocity encoding sensitive to arterial blood flow speed to study the relation between the slope of the cardiac pulse in the cerebral arteries and episodic memory (Vikner et al., 2021). They found that steeper systolic onsets correlated with poorer episodic memory performance. The ability of our technique to extract the shape of the cardiac pulse, including slope and width, in both blood vessels and CSF spaces may help further elucidate these types of effects.

The cardiac aligned responses are not well described by a sinusoidal oscillation, but show a particular temporal asymmetry that matches known aspects of cerebral fluid circulation. The responses in the anterior cingulate and sagittal sinus that we observed drop sharply around the onset of the PPG peak, followed by a slow rise. Blood flow measurements in other studies from slices that include the carotid artery (Enzmann et al., 1994; Wagshul et al., 2011) show a sharp rise in the speed of flow related to systole that aligns with the sharp drop in our data (Figure 3d, top panel).

The lateral ventricles show a signal change in the opposite direction, with a local maximum at the time of the PPG peak. The fact that an area close to a cerebral artery and the superior sagittal sinus behave in an opposite manner compared to CSF can be explained by the fact that the cranium is an enclosed space. Within this enclosed space, systole generates a rapid increase in blood pulsing into the cerebral arteries, which displaces CSF (Wagshul et al., 2006; Wagshul et al., 2011). Whereas other methods have characterized these interactions across several seconds during sleep (Fultz et al., 2019), this method reveals these interactions at the timescale of a few hundreds of milliseconds within a heartbeat cycle.

### 4.4 Implications for resting‐state fMRI analyses

Previous studies have emphasized the importance of understanding the effects of cardiac pulsations on resting‐state fMRI networks (Bayrak et al., 2021; Chen et al., 2020; Shmueli et al., 2007). One study examined the effects of changes in heart rate on the fMRI response (Chang et al., 2009) and identified a heart rate response function that evolves over a few seconds with changes in heart rate. Although we are interested in these heartbeat related signals, these resting‐state studies emphasize the removal of cardiac effects (Glover et al., 2000; Hu et al., 1995). These effects can be significant and hard to remove because cardiac pulsations influence fMRI signals in brain regions within a second, but fMRI data are typically sampled every 1–2 s. This undersampling results in aliasing of heartbeat related signals. Two studies have shown that faster measurements can reduce the size of these unwanted signals (Huotari et al., 2019; Jahanian et al., 2019).

There are many large resting‐state fMRI datasets with TRs of 1–2 s. Our slow fMRI analyses demonstrate that such measurements can be, retrospectively, aligned when a pulse oximetry measurement is available. The cardiac aligned responses could be used in a forward manner to correct for unwanted heartbeat driven fluctuations in the resting‐state signal.

We used a relatively common sequence to estimate the cardiac pulse waveforms. Other rapid MRI sequences have been designed to sample BOLD signals at fast rates, including multi‐slab echo‐volumar imaging, (Posse et al., 2012, 2013), ultrafast generalized inverse imaging (Boyacioglu et al., 2013), magnetic resonance inverse imaging (Lin et al., 2012), and MREG (Lee et al., 2013). These rapid sequences facilitate filtering out physiological noise and estimating the BOLD signal in resting‐state networks. Rather than filtering out the physiological signals, we show that common sequences can also characterize the cardiac pulse pressure waves, which opens up applications across cognitive neuroscience studies that use resting‐state fMRI measurements.

### 4.5 Study limitations

This article develops methodology to characterize the cardiac waveform in cranial blood and CSF spaces in five subjects. While this is a small sample size, it demonstrates the reliability of the method across a highly variable age range from 24 to 63 years. Future studies will now be able to use the quantitative outputs from this method (peak time, sign, and width) to study intersubject variations.

There are several additional measurements that can be done to better understand how pressure, flow speed, flow volume and motion affect the observed pulsations. Blood flow in the brain is affected by arterial blood pressure (influencing blood flow into the brain) and venous sinus pressure (influencing blood return to the heart, venous sinus pressure is approximately equal to the intracranial pressure) (Lassen & Christensen, 1976; Ruesch et al., 2021). Blood pressure measurements could help understand differences across subjects in the percent signal change related to the arterial vasodilation and constriction. Moreover, blood pressure is known to alter pulse transit times, and could explain differences in arterial–venous phase delays between the subjects.

Finally, this method quantifies cardiac pulsatile waveforms by using the fact that every slice is acquired within 50 ms. The next sample of the voxel is measured one repetition time (250 ms) later. The method relies on the idea that the measured waveforms are consistent from beat to beat. This consistency is confirmed by the test–retest reliability. Extracting between‐beat changes in the waveform, perhaps related to breathing or vasomotion, will need either different analyses or different scan protocols that measure at even higher temporal sampling (Dreha‐Kulaczewski et al., 2015; Posse et al., 2013).

### 4.6 Conclusion

Cardiovascular mechanisms are essential for healthy cognitive and affective function. Neurological diseases and aging affect the way in which the cardiac pulse affects the brain's fluid dynamics. To understand whether fMRI data can help assess the spatial distribution and temporal delays of the cardiac pulsations, and conversely, how cardiac pulsations affect the fMRI signal, we collected data with a fast SMS sequence and, retrospectively, aligned the measurements to heartbeats. Cardiac aligned responses reveal the combined impact of the cardiac pulse pressure wave and blood flow on the fMRI signal during the cardiac cycle. Identifying the typical range of responses in the healthy may help us identify atypical responses in neurological and psychiatric diseases.

## Supporting information


**APPENDIX S1** Supplemental Materials

**MOVIE S1** Supplementary Movie

**MOVIE S2** Supplementary Movie

## Data Availability

All our methods are open‐source and shared on GitHub (https://github.com/vistalab/BrainBeat). Data are available in BIDS format on OpenNeuro.org under (doi:10.18112/openneuro.ds004213.v1.0.0).
