# Supplementary material for: Measuring brain beats: Cardiac‐aligned fast functional magnetic resonance imaging signals
Source: Hum Brain Mapp. 2022 Oct 29;44(1):280–94. doi: 10.1002/hbm.26128 (PMC9783469; doi:10.1002/hbm.26128)
Supplement: Supplementary file 1 — APPENDIX S1 Supplemental Materials [file HBM-44-280-s001.pdf]

Supplemental Materials

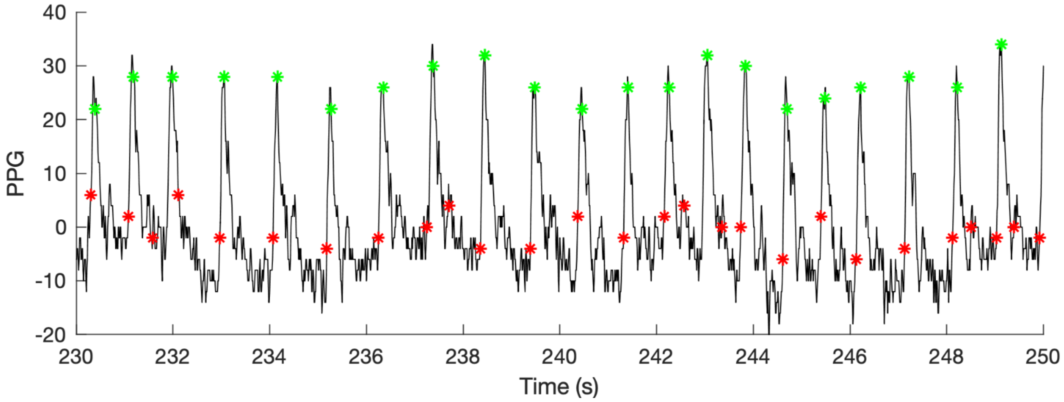

Supplemental Figure 1. Detection of heartbeats in the raw PPG signal shown as a function of time (seconds). Red dots show the heart beats detected by the GE scanner software in real time, note that there are several false positive peaks detected and a few peaks are missed. Green dots show the heart beats detected by our algorithm (section 2.3). Peaks are detected in the low pass filtered signal, and green dots sometimes appear a few samples to the left or right of the peak, but there were fewer false positives.

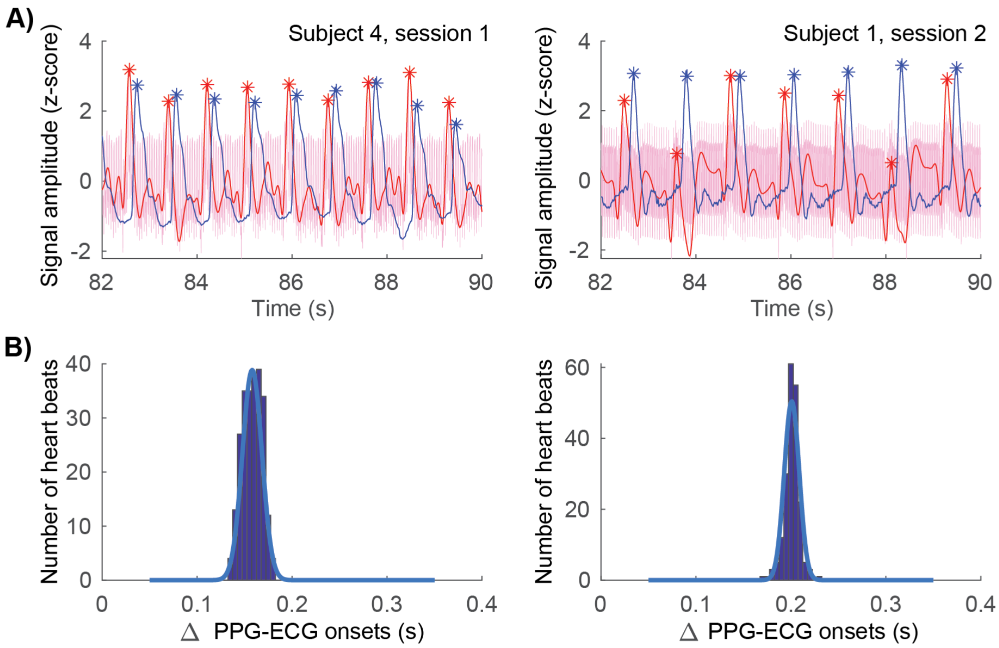

Supplemental Figure 2. ECG vs PPG peak detection. A) Segment of raw PPG and ECG traces in two subjects where we collected ECG data in addition to PPG data. Blue trace shows the PPG signal, with an asterisk indicating the PPG peak. The pink trace shows the raw ECG data that were strongly contaminated by scanner noise. The red trace is the low pass filtered ECG signal, with an asterisk indicating the ECG peak. B) Histograms of the differences (Δ) between the peaks detected in the ECG and PPG were highly consistent across hear beats, with 95% confidence intervals of the average difference ranging from 0.157-0.159 ms and 0.200-0.202 ms. This indicates that **while using ECG vs PPG signals for detecting pulse peaks may shift all waveforms in time,** the timing of the pulse peak can be accurately detected with PPG and **the overall shape will remain the same.**

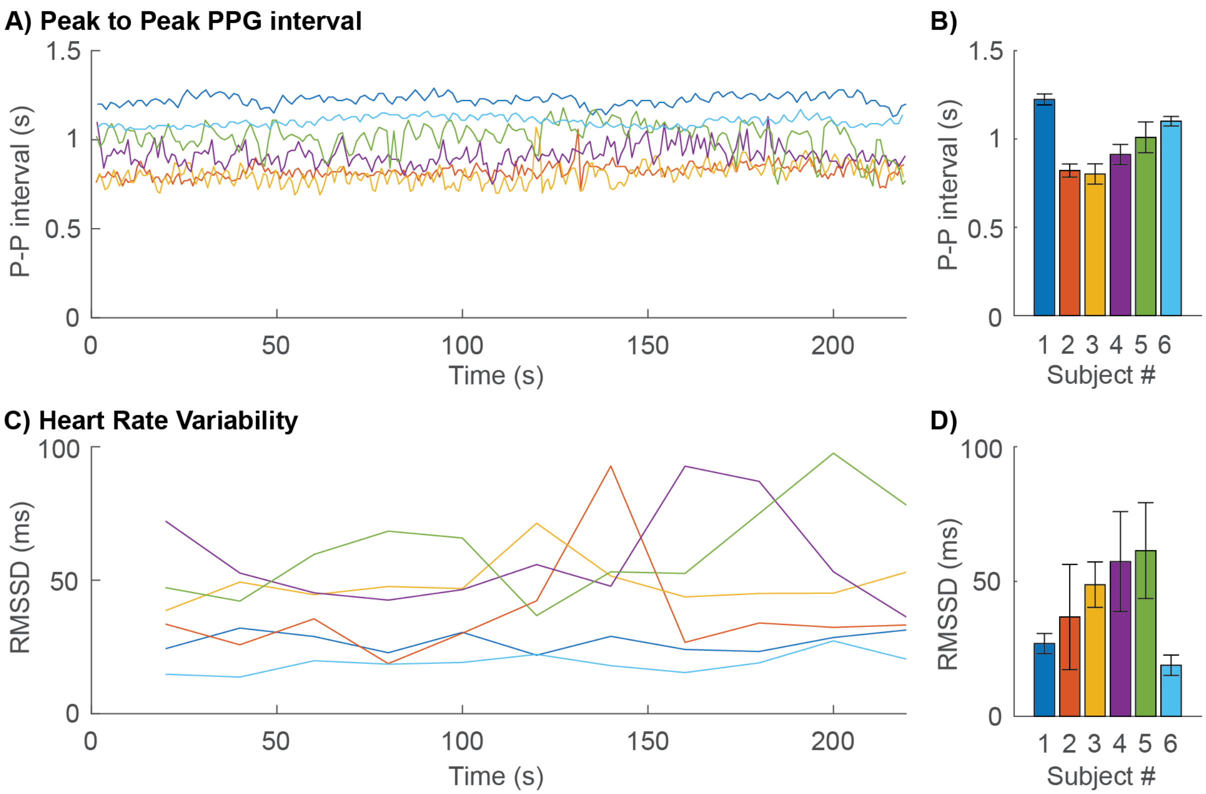

Supplemental Figure 3. Heart Rate Variability (HRV). A) Peak to peak intervals (P-P intervals) in seconds between subsequent PPG peaks plotted in a different color for each subject. B) Corresponding bar graphs of the P-P intervals +/- one standard deviation. C) Heart rate variability was calculated across ultra-short intervals of 20 seconds using the Root Mean Square of Successive Differences (RMSSD) (Salahuddin et al. 2007). Each value is plotted at the time point at the end of the interval. D) The average RMSSD for each subject +/- one standard deviation shows values within a normal range (Shaffer and Ginsberg 2017).

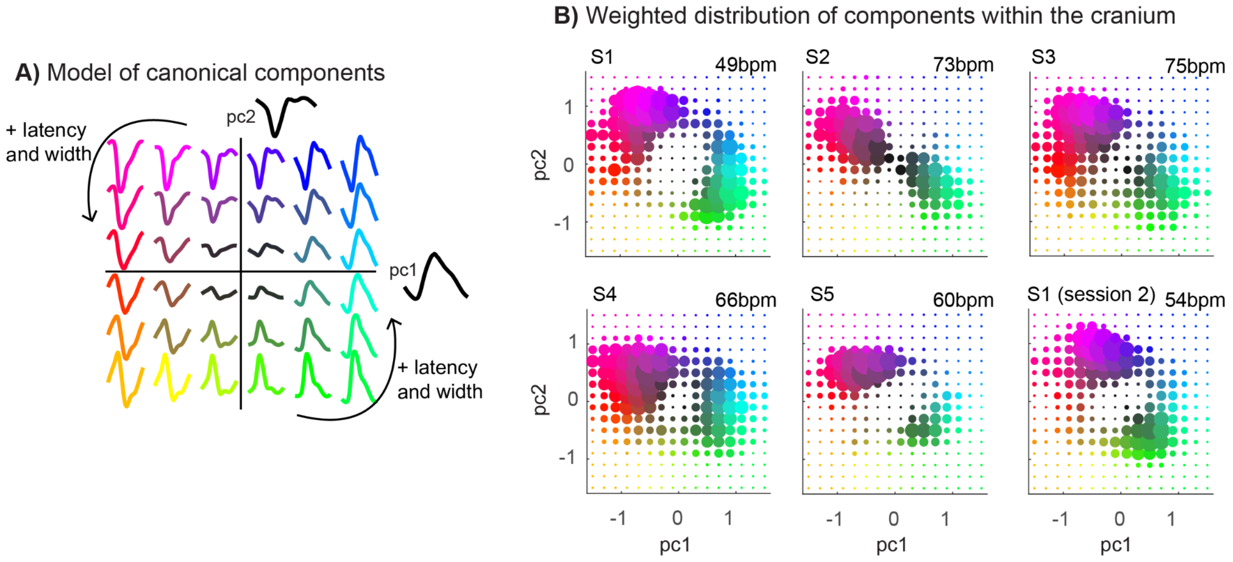

Supplemental Figure 4. Modeling the cardiac aligned responses. A) Canonical components (pc1 and pc2) across the subjects can be combined with various weights to predict different cardiac aligned response shapes. **D)** Fitting these two canonical components in each subject shows that component weights cluster into two groups with low pc1, high pc2 weights (purple - red) and with high pc1, low pc2 weights (green - blue). The dot size indicates the number of voxels with component weights. Only highly reliable voxels with R^2>70 were included.

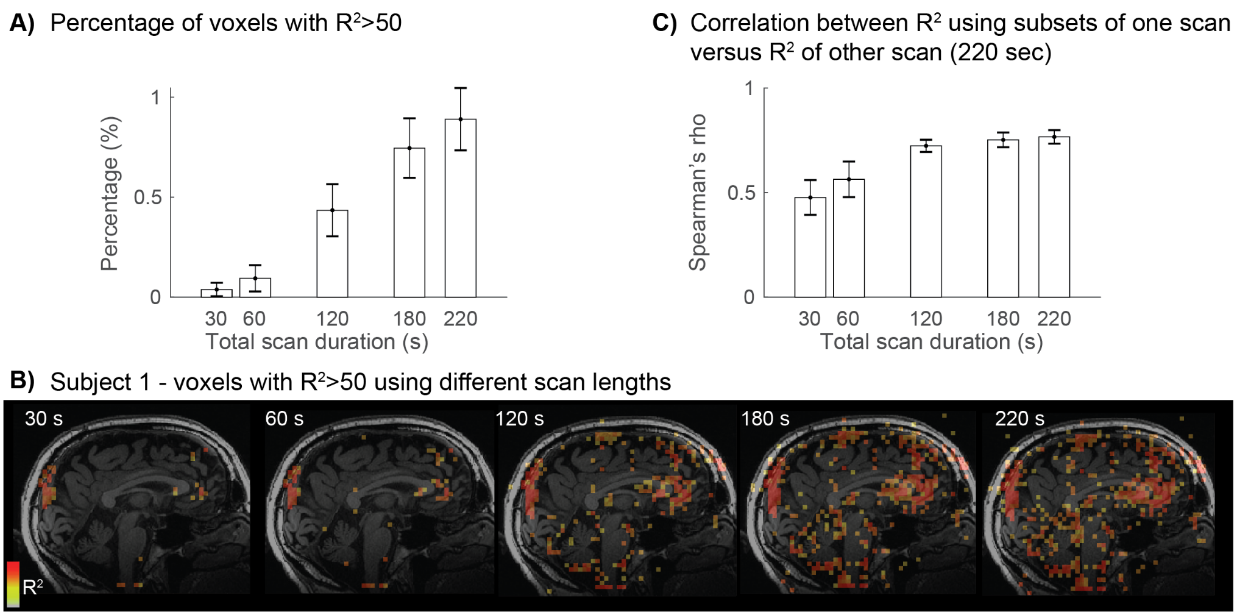

Supplemental Figure 5. Investigation of different scan durations. A) As an indication of the number of areas where we calculated the cardiac pulsations with less data. The total scan duration was 220 seconds, and we also used 30, 60, 120, 180 sec of data to calculate the percentage of voxels with reliable heart beat aligned responses. Reliable responses were considered those voxels with an **R^2>50** between even and odd responses. Bars show the average across subjects +/- one standard deviation across the subjects. **B)** Example of the voxels with reliable cardiac pulsations (**R^2>50**) calculated based on different scan length overlaid on a T1 image in one subject. It can be seen that with scan durations shorter than 120 seconds, many regions are missed at this threshold. **C)** To understand whether a shorter scan duration result in different patterns of reliable cardiac pulsations, or whether the reliability simply decreases while the overall pattern remains the same, we calculated the spatial correlation (Spearman's rho) between the R^2 calculated based on a subsample of one scan versus the R^2 calculated on a separate 220 sec long scan. The y-axis shows the average across subjects +/- one standard deviation. This suggests that with shorter scan durations, similar spatial patterns of reliable cardiac pulsations will be found when scanning for 120 seconds or longer, but cardiac aligned response shapes will be more reliable with longer scan durations (calculated up to 220 sec). We note that longer scan durations may increase chances of subject motion and lower reliability.

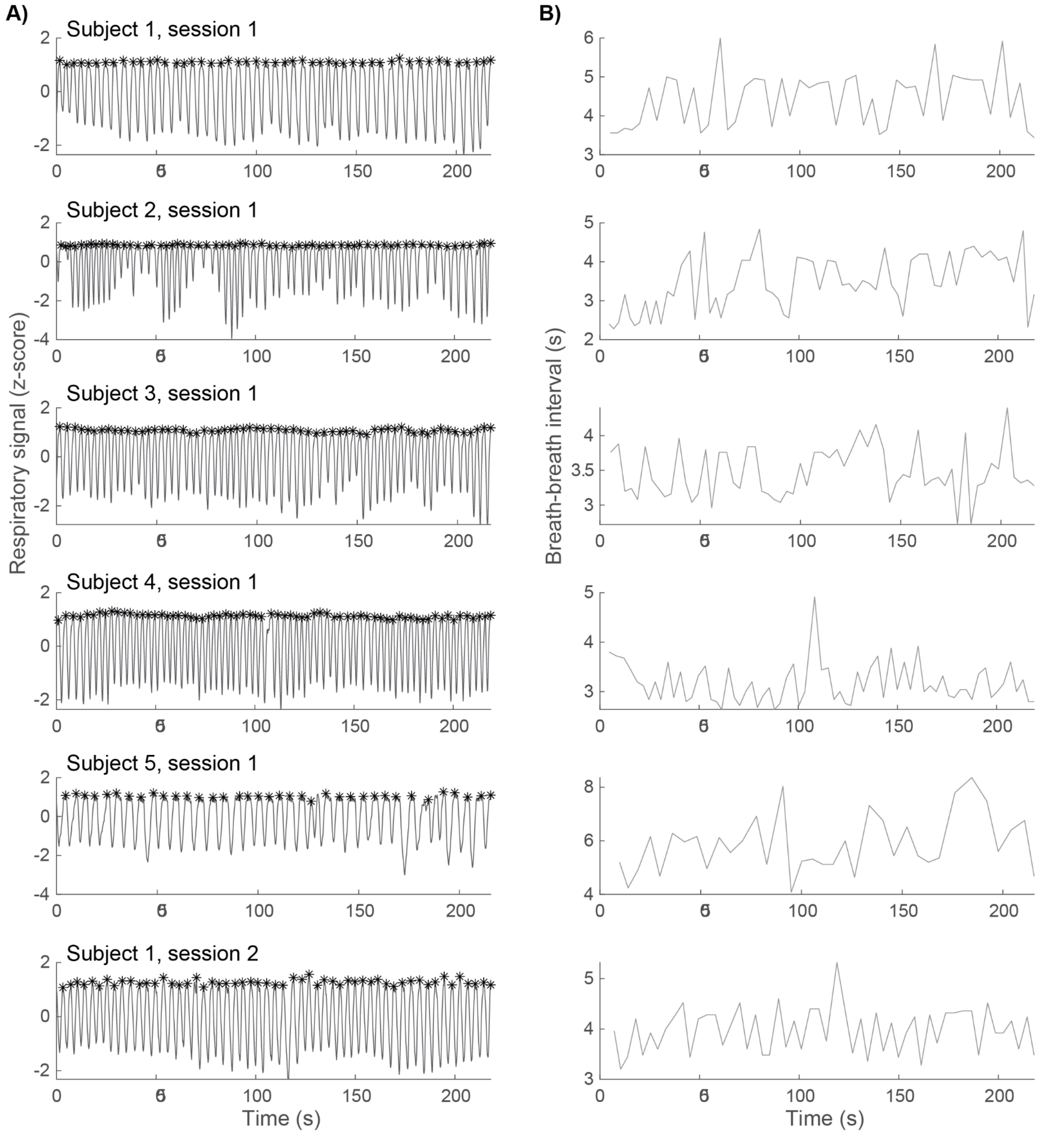

Supplemental Figure 6. Respiration rate. A) During all scans, respiration was measured with a breathing belt. The z-scored signal is plotted with the detected peaks indicated with an asterisk. B) From the detected peaks we calculated respiration-respiration intervals (in seconds) across the data acquisition.
